# Molecular and Functional Characterization of BDNF-Overexpressing Human Retinal Pigment Epithelial Cells Established by *Sleeping Beauty* Transposon-Mediated Gene Transfer

**DOI:** 10.3390/ijms232112982

**Published:** 2022-10-26

**Authors:** Larissa Mattern, Katrin Otten, Csaba Miskey, Matthias Fuest, Zsuzsanna Izsvák, Zoltán Ivics, Peter Walter, Gabriele Thumann, Sandra Johnen

**Affiliations:** 1 Department of Ophthalmology, University Hospital RWTH Aachen, 52074 Aachen, Germany; 2 Division of Medical Biotechnology, Paul-Ehrlich-Institute, 63225 Langen, Germany; 3 Max Delbrück Center for Molecular Medicine in the Helmholtz Association, 13125 Berlin, Germany; 4 Department of Ophthalmology, University Hospitals of Geneva, 1205 Geneva, Switzerland; 5 Experimental Ophthalmology, University of Geneva, 1205 Geneva, Switzerland

**Keywords:** RPE cells, BDNF, *Sleeping Beauty* transposon system, non-viral transfection, cell-based additive gene therapy, neurodegenerative retinal diseases, AMD

## Abstract

More and more patients suffer from multifactorial neurodegenerative diseases, such as age-related macular degeneration (AMD). However, their pathological mechanisms are still poorly understood, which complicates the development of effective therapies. To improve treatment of multifactorial diseases, cell-based gene therapy can be used to increase the expression of therapeutic factors. To date, there is no approved therapy for dry AMD, including late-stage geographic atrophy. We present a treatment option for dry AMD that transfers the brain-derived neurotrophic factor (*BDNF*) gene into retinal pigment epithelial (RPE) cells by electroporation using the plasmid-based *Sleeping Beauty* (*SB*) transposon system. ARPE-19 cells and primary human RPE cells were co-transfected with two plasmids encoding the *SB100X* transposase and the transposon carrying a *BDNF* transcription cassette. We demonstrated efficient expression and secretion of BDNF in both RPE cell types, which were further increased in ARPE-19 cell cultures exposed to hydrogen peroxide. BDNF-transfected cells exhibited lower apoptosis rates and stimulated neurite outgrowth in human SH-SY5Y cells. This study is an important step in the development of a cell-based *BDNF* gene therapy that could be applied as an advanced therapy medicinal product to treat dry AMD or other degenerative retinal diseases.

## 1. Introduction

Our ageing society increasingly battles neurodegenerative diseases that are complex and difficult to treat. Age-related macular degeneration (AMD) affects people over the age of 60 and impairs the central retina, leading to severe vision loss. It is considered the leading cause of blindness, and meta-analyses have shown that AMD is expected to increase to 288 million patients worldwide by 2040 [1] and will affect 77 million people in Europe by 2050 [2]. AMD is multifactorial, with age, smoking, and genetic predisposition being identified as risk factors. Clinically, three stages are distinguished: early, intermediate, and late (advanced) AMD, which is divided into geographic atrophy (GA, dry AMD) and choroidal neovascularization (CNV, wet/neovascular AMD). Wet AMD accounts for a much smaller percentage of the disease and is reasonably treatable by repeated intravitreal injections of anti-VEGF drugs [3]. In contrast, dry AMD, which is characterized by progressive atrophy of photoreceptor cells, retinal pigment epithelium (RPE) cells, and choriocapillaris, has no approved therapy to date, but several therapies being clinically tested at the moment. These clinical trials involve the complement system using the C3 inhibitor pegcetacoplan [4] or the C5 inhibitor avacincaptad pegol [5], the visual cycle using the deuterated vitamin A derivate ALK-001 [6], cell-based therapies using human embryonic stem cell-derived RPE cells [7,8], and neuroprotection using elamipretide [9,10].

In addition to the above clinical trials, gene therapy is another promising approach to treat retinal diseases. As the eye is a small, enclosed organ with a blood-retinal barrier, small amounts of genetic material can be administered into the eye without triggering a systemic immune response. The first gene therapy for ophthalmology was approved in 2017: voretigene neparvovec-rzyl (Luxturna^®^) is used to treat retinitis pigmentosa patients with a bi-allelic mutation in the *RPE65* gene [11,12]. To treat advanced AMD, several gene therapeutics are currently in clinical trials: GT005 [13] is used for the treatment of GA and is a recombinant adeno-associated viral (AAV) vector that encodes human complement factor I (ClinicalTrials.gov identifier: NCT03846193, NCT04437368, and NCT04566445); AAVCAGsCD59 is tested for both dry and wet AMD (NCT03144999 and NCT03585556) and expresses a soluble form of the membrane regulatory protein CD59. Viral vectors encoding other anti-angiogenic factors are also being tested for the treatment of wet AMD. These include RGX-314 (NCT03066258, NCT03999801, NCT04514653, NCT04704921, and NCT04832724), ADVM-022 (NCT03748784 and NCT04645212), and RetinoStat (NCT01301443 and NCT01678872) [14].

Advanced neovascular AMD has also been treated with a single intravitreal injection of a non-integrating adenoviral (Ad) vector encoding pigment epithelium-derived factor (PEDF) [15]. Data from this phase I clinical trial indicated that high-dose administration of AdPEDF.11 resulted in anti-angiogenic activity that persisted for several months without causing severe inflammation or serious adverse events. PEDF belongs to the family of non-inhibitory serpins and was purified for the first time from the conditioned medium of human RPE (hRPE) cells [16,17]. It is a multifunctional protein that has anti-angiogenic, anti-oxidative, and neurotrophic properties, among others [18].

On the basis of the above data, we previously developed a cell-based gene therapy approach in which the human *PEDF* gene is introduced ex vivo into pigment epithelial cells [19]. Our aim was to use these genetically modified cells as a continuous source of PEDF to generate an enhanced anti-angiogenic milieu. Our subsequent in vitro and in vivo studies have shown that transplantation of these PEDF-secreting cells into the eye significantly reduced pathological endothelial cell growth and neovascularization induced by laser or alkali burn [20,21,22]. Genes were introduced into the pigment epithelial cells by electroporation using the *Sleeping Beauty* (*SB*) transposon system [23], and protein secretion was found to be sustained and stable [24,25,26]. The two-component *SB* transposon system is transferred as a plasmid construct, making its manufacturing simple and inexpensive. The use of the *SB* transposon system supports stable integration and long-term expression of the therapeutic gene. In addition, it enables genomic insertion of transgenes in an almost random manner [27], which enhances the safety of this system in human applications.

Our current study uses brain-derived neurotrophic factor (BDNF), rather than PEDF. BDNF belongs to the nerve growth factor (NGF) superfamily along with NGF, neurotrophin-3, and neurotrophin-4/5, and exerts its effects through the high affinity receptor tropomyosin-related kinase B (TrkB) and the common low affinity receptor p75 neurotrophin receptor (p75NTR) [28]. BDNF is highly expressed in the brain [29,30,31,32] and is also detectable in the eye [33,34], where it is produced in retinal ganglion cells (RGCs), amacrine cells, and Müller cells [35,36]. The initially synthesized pre-pro-BDNF precursor is processed to pro-BDNF (~32 kDa), whose cleavage of the N-terminal pro-domain releases the mature BDNF (~13 kDa) [37]. BDNF affects neuronal development, growth, and survival and is important for neurogenesis, synaptogenesis, and synaptic plasticity [38,39]. Decreased BDNF levels are associated with Parkinson’s (PD) and Alzheimer’s (AD) diseases [40,41] and have also been detected in the serum, aqueous humor, and lacrimal fluid of patients with diabetic retinopathy, AMD, and glaucoma [42,43,44]. In vivo models of AD, acute optic nerve injury, and experimental glaucoma demonstrated the neuroprotective effects of virally administered BDNF by attenuating behavioral deficits, preventing neuron loss, and increasing RGC survival, respectively [45,46,47,48]. Most recently, AAV2-based *BDNF* gene therapy has been used in a phase I clinical trial to investigate whether it can slow or prevent cell loss in the brain of patients with AD and mild cognitive impairment (NCT05040217).

Based on the previously established cell-based *PEDF* gene therapy, we have developed a protocol for efficient transfer of the human *BDNF* gene into the RPE cell line ARPE-19 and into primary hRPE cells using the *SB* transposon system. We demonstrate that the genetic modification is efficient and stable, and that the transfected cells secrete sufficient amounts of BDNF, resulting in a reduced apoptosis rate and inducing neurite outgrowth in co-cultured SH-SY5Y cells. In addition, transfected ARPE-19 cells exposed to hydrogen peroxide (H_2_O_2_) showed more *BDNF* expression and BDNF secretion than cells not exposed to H_2_O_2_.

## 2. Results

### 2.1. Sleeping Beauty Transposon-Mediated Delivery of the Human BDNF Gene into the ARPE-19 Cell Line

**Figure 1:**
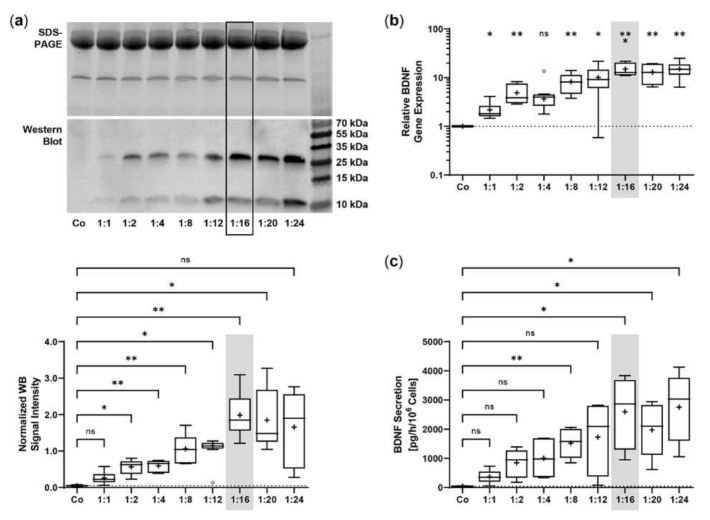
Transfection of ARPE-19 cells using different ratios of *SB100X* transposase plasmid and *BDNF* transposon plasmid. In each experiment (*n* = 6), 1 × 10^5^ cells were transfected with an increasing ratio (1:1–1:24) of *SB100X* transposase to *BDNF* transposon plasmid at a total concentration of 0.5 µg, plus one control without the addition of plasmid DNA (Co). Cultures were terminated 7.33 ± 0.52 days after transfection. Cells were used for the isolation of total RNA. Medium used for western blot and ELISA was added to the cells in a defined volume 24 h before culture termination. (**a**) BDNF secretion was analyzed by western blots of the culture medium. Loading of equal amounts of supernatant was proven by Stain-Free imaging technology. Each western blot signal was referenced to the total protein content of the sample and normalized to the mean value of the respective experiment. BDNF signals were more evident in transfected cultures than in Co cultures (ns: not significant, * *p* < 0.05, ** *p* < 0.01, Brown-Forsythe and Welch ANOVA tests with Dunnett’s T3 multiple comparisons test). (**b**) *BDNF* gene expression was analyzed by qPCR. Total (endogenous + recombinant) *BDNF* expression in transfected cultures was related to the expression in Co cultures, which was set to 1 (dashed line). An increase in *BDNF* gene expression was observed in the transfected cultures (ns: not significant, * *p* < 0.05, ** *p* < 0.01, *** *p* = 0.0008, one sample t test). (**c**) BDNF secretion was quantified by ELISA using precisely defined cell culture supernatants. Values of the transfected cultures were higher than those of the Co cultures (ns: not significant, * *p* < 0.05, ** *p* = 0.004, Brown-Forsythe and Welch ANOVA tests with Dunnett’s T3 multiple comparisons test). Data are presented as box and whisker plots (whiskers: minimum to maximum, mean values indicated by +). Outliers were evaluated using the ROUT method (Q = 1%, indicated by red open circles).

The *SB100X* transposase and *BDNF* transposon were provided on separate plasmids to regulate and optimize the transposase-to-transposon ratio during electroporation. Optimal transposition efficiency was determined based on *BDNF* gene expression and BDNF protein secretion for plasmid ratios ranging from 1:1 to 1:24 at a constant total plasmid amount of 0.5 µg. BDNF was detected by immunoblotting in its glycosylated precursor form (pro-BDNF, ~32 kDa) and in the post-translationally cleaved mature form (~13 kDa, Figure 1a). Signal intensities increased with increasing plasmid ratios, starting at 0.05 ± 0.02 for the non-transfected ARPE-19 cultures and 0.26 ± 0.17 to 0.98 ± 0.42 for ratios of 1:1 to 1:12. The ratios 1:16 to 1:24 had the highest signal intensities, which were 7.6, 7.1, and 6.4 times higher, respectively, than the value of the ratio 1:1 (Figure 1a). This gradual increase was also observed at the level of gene expression. Starting from a value of 2.17 ± 0.98 at the 1:1 ratio, the highest *BDNF* expression levels were seen at ratios of 1:16 to 1:24. Here, relative *BDNF* expression was increased 6.9-, 6.0-, and 7.0-fold, respectively (Figure 1b). ELISA-based quantification revealed a baseline secretion of 44.8 ± 14.2 pg BDNF/h/10^6^ cells in non-transfected ARPE-19 cultures. Transfection with a 1:1 plasmid ratio resulted in an 8.3-fold higher BDNF secretion (370.5 ± 240.1 pg/h/10^6^ cells), which further increased with increasing plasmid ratios (1728 ± 1225 pg/h/10^6^ cells at a 1:12 ratio). Highest BDNF secretion rates were observed in cultures transfected with plasmid ratios ranging from 1:16 to 1:24: they showed 7.0-, 5.3-, and 7.4-fold higher secretion compared with cultures transfected with the 1:1 ratio (Figure 1c). Because the highest expression and secretion levels and, more importantly, the least variation were observed at a ratio of 1:16, all subsequent ARPE-19 transfections were performed with this ratio, corresponding to 0.03 µg of *SB100X* transposase plasmid and 0.47 µg of *BDNF* transposon plasmid.

**Figure 2:**
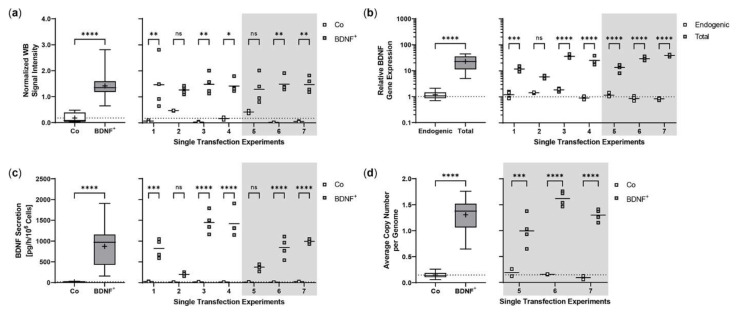
Transposition efficiency in ARPE-19 cells transfected with a defined ratio of *SB100X* transposase to *BDNF* transposon plasmid. In each experiment (*n* = 7), four transfections were performed using 5 × 10^4^ cells and a mixture of 0.03 µg *SB100X* transposase and 0.47 µg *BDNF* transposon plasmid (BDNF^+^), plus two controls without the addition of plasmid DNA (Co). Cultures were terminated 21.0 ± 0.58 days after transfection. Cells were used for isolation of total RNA and genomic DNA, respectively. Medium used for western blot and ELISA was added to the cells in a defined volume 24 h before culture termination. (**a**) BDNF secretion was analyzed by western blots of culture medium using Stain-Free Gels. Each western blot signal was referenced to the total protein content of the sample and normalized to the mean value of the respective experiment. Signals in BDNF^+^ cultures were increased compared to Co cultures (ns: not significant, summarized data: **** *p* < 0.0001, unpaired t test with Welch’s correction; single experiments: * *p* = 0.0127, ** *p* < 0.01, 2way ANOVA with Šídák’s multiple comparisons test). (**b**) *BDNF* gene expression was analyzed by qPCR. Endogenous and total *BDNF* expression in BDNF^+^ cultures were related to the respective expression in Co cultures, which were set to 1 (dashed line). An increase in total *BDNF* expression was observed in all BDNF^+^ cultures (ns: not significant, summarized data: **** *p* < 0.0001, unpaired t test with Welch’s correction; single experiments: *** *p* = 0.0009, **** *p* < 0.0001, 2way ANOVA with Šídák’s multiple comparisons test). (**c**) BDNF secretion was quantified by ELISA using precisely defined cell culture supernatants. Values of the BDNF^+^ cultures were higher than those of the Co cultures (ns: not significant, summarized data: **** *p* < 0.0001, unpaired t test with Welch’s correction; single experiments: *** *p* = 0.0002, **** *p* < 0.0001, 2way ANOVA with Šídák’s multiple comparisons test). (**d**) *BDNF* transposon copy numbers were determined by ddPCR. Average copy numbers per diploid genome in BDNF^+^ cultures were significantly higher compared to the Co cultures (ns: not significant, summarized data: **** *p* < 0.0001, unpaired t test with Welch’s correction; single experiments: *** *p* = 0.0007, **** *p* < 0.0001, 2way ANOVA with Šídák’s multiple comparisons test). Data are presented as box and whisker plots (whiskers: minimum to maximum, mean values indicated by +) and as individual values per experiment (line at mean).

Repeated experiments were performed with an initial number of 5 × 10^4^ ARPE-19 cells. Cultures were terminated 21.0 ± 0.58 days after transfection to determine *BDNF* gene expression and BDNF protein secretion in each non-transfected and transfected approach. Western blot analysis showed an 8.0-fold increase in BDNF signal intensity in transfected cells compared to non-transfected cells. Highest variation was observed in the first experiment with minimum and maximum values of 0.65 and 2.81, respectively (Figure 2a). At transcriptional level, transfected cells showed a 19.4-fold increase in total *BDNF* expression compared to endogenous *BDNF* expression. The largest differences between total and endogenous *BDNF* gene expression were observed in experiments 3, 4, 6, and 7 (Figure 2b). ELISA-based quantification of BDNF secretion was consistent with the results observed for *BDNF* gene expression. Transfected cells secreted an average of 871.9 ± 483.8 pg BDNF/h/10^6^ cells, a 48.0-fold increase compared to 18.17 ± 7.189 pg/h/10^6^ cells in non-transfected cells. The highest increase in BDNF secretion was observed in experiments 3, 4, and 6; however, the largest differences between individual transfections within an experiment were also found here (Figure 2c). The average copy number of the *BDNF* transgene in the transfected ARPE-19 cultures was 1.31 ± 0.32 compared to the ddPCR-derived estimate of 0.15 ± 0.07 in non-transfected cultures and ranged from 0.58 copies in experiment 5 to 1.87 copies in experiment 6 (Figure 2d).

### 2.2. Proliferation and Cell Viability of BDNF-Transfected ARPE-19 Cell Cultures

**Figure 3:**
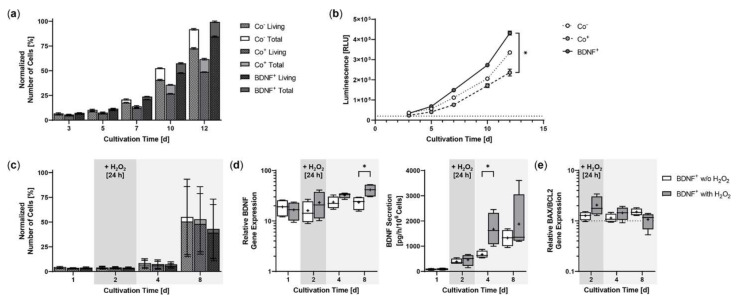
Growth behavior of BDNF-transfected ARPE-19 cells without or after H_2_O_2_-induced oxidative stress compared to non-transfected control cells. In each experiment (*n* = 3 for cultures without H_2_O_2_ exposure, *n* = 4 for cultures with H_2_O_2_ exposure), non-treated cells (Co^−^), cells exposed to the electric field without plasmid DNA (Co^+^), and BDNF-transfected cells (BDNF^+^, transfected with 0.03 µg *SB100X* transposase and 0.47 µg *BDNF* transposon plasmid) were seeded at an initial density of 2 × 10^4^ cells. (**a**) Without H_2_O_2_ exposure, individual cultures were terminated after 3, 5, 7, 10, and 12 days. Proliferation was determined by classifying live cell count and total cell count. All values were normalized to the highest measured value, which was set to 100%. At the end of the experiment, comparison of the number of living cells or total cells showed no significant differences, and within each culture, the differences between live cell count and total cell count were also not significant (Kruskal-Wallis test with Dunn’s multiple comparison test). (**b**) Cell viability was analyzed using a luminescence-based assay. At the end of the experiment, BDNF^+^ cultures showed significantly higher values than the Co^+^ cultures (* *p* = 0.0219, Kruskal-Wallis test with Dunn’s multiple comparisons test). (**c**) In the case of H_2_O_2_ treatment, individual cultures were terminated after 1, 2, 4, and 8 days. Cells were used for counting and for isolation of total RNA. Medium used for ELISA was added to the cells in a defined volume 24 h before culture termination. No significant differences in cell number were observed at the end of the experiment (Kruskal-Wallis test with Dunn’s multiple comparison test). Data are presented as mean ± SD. (**d**) *BDNF* gene expression was analyzed by qPCR. Total *BDNF* expression in BDNF^+^ cultures without and with H_2_O_2_ exposure was related to the expression in the corresponding Co cultures, which was set to 1. Increased *BDNF* expression was observed in all transfected cultures (* *p* < 0.05 for BDNF^+^ w/o H_2_O_2_ and with H_2_O_2_ at days 1 and 2. BDNF secretion in BDNF^+^ cultures was quantified by ELISA using precisely defined cell culture supernatants. Comparison of BDNF^+^ cultures at each culture termination showed significantly increased *BDNF* expression and significantly increased BDNF secretion for cultures with H_2_O_2_ at day 8 and day 4, respectively (* *p* < 0.05, Mann-Whitney test). (**e**) *BAX* and *BCL2* gene expression were analyzed by qPCR. The *BAX*/*BCL2* expression ratios in BDNF^+^ cultures without and with H_2_O_2_ exposure were related to the expression ratios in the corresponding Co cultures, which were set to 1. An increased ratio of *BAX*/*BCL2* expression was observed in the transfected cultures. Comparison of BDNF^+^ cultures without and with H_2_O_2_ exposure at each termination date showed no significant differences (Mann-Whitney test). Data are presented as box and whisker plots (whiskers: minimum to maximum, mean values indicated by +).

Starting from an initial seeding of 2 × 10^4^ ARPE-19 cells, BDNF^+^ cultures showed the strongest proliferation rate during a cultivation period of 12 days. After three days, the number of living cells in the BDNF^+^ cultures were 10.8% higher than in the non-transfected ARPE-19 cultures (Co^−^) and 36.7% higher than in the cultures exposed only to the electric field (Co^+^). At the end of cultivation, the difference was 16.5% and 73.5%, respectively, and the percentage of living cells relative to total cells in the different cultures was 79.0% for the two control cultures and 85.0% for the BDNF^+^ cultures (Figure 3a). A luminescence-based viability assay also showed the highest number of living cells in the BDNF^+^ cultures. After 12 days, the relative luminescence of BDNF^+^ cultures was 1.3- and 1.8-fold higher than that of Co^−^ cultures and Co^+^ cultures, respectively, whose values were again 1.4-fold lower than those of Co^-^ cultures (Figure 3b). Treatment with 150 µM H_2_O_2_ for 24 h resulted in a different proliferation behavior. Immediately after H_2_O_2_ exposure, the number of living cells was 4.7% to 7.7% lower in BDNF^+^ cultures than in control cultures. After eight days, the difference increased to 18.5% and 22.3% compared to Co^+^ and Co^-^ cells, respectively. The percentage of living cells relative to total cells in the different cultures ranged from 90.9% to 91.5% (Figure 3c). Comparison of *BDNF* expression in BDNF^+^ cultures without and with H_2_O_2_ treatment showed increasing values for cultures with H_2_O_2_, ranging from a ratio of 1.4 at day 2 (16.04 ± 7.99 w/o H_2_O_2_ versus 23.16 ± 13.86 with H_2_O_2_) to 1.8 at day 8 (23.41 ± 7.09 versus 41.40 ± 12.19, Figure 3d). BDNF secretion was 386.2 ± 113.6 pg/h/10^6^ cells at day 2 and increased to 668.3 ± 145.9 at day 4 and 1324 ± 326.8 at day 8 for BDNF^+^ cultures without H_2_O_2_ and from 458.0 ± 226.7 pg/h/10^6^ cells to 1672 ± 643.7 and 1875 ± 1155 for BDNF^+^ cultures with H_2_O_2_, corresponding to 1.2-, 2.5-, and 1.4-fold higher BDNF secretion, respectively, for cultures after H_2_O_2_ exposure (Figure 3d). ARPE-19 control cultures secreted an average of 23.65 ± 17.41 pg BDNF/h/10^6^ cells (data not shown). H_2_O_2_ treatment also altered the *BAX*/*BCL2* expression ratio in the BDNF^+^ cultures: immediately after treatment, it increased to 2.06 and was 1.6-fold higher than in the cultures without H_2_O_2_ exposure. Subsequently, the *BAX*/*BCL2* expression ratio decreased in the cultures with H_2_O_2_ exposure (1.44 at day 4, 1.07 at day 8), whereas it increased in the cultures without H_2_O_2_ exposure (1.14 at day 4, 1.51 at day 8, Figure 3e).

**Figure 4:**
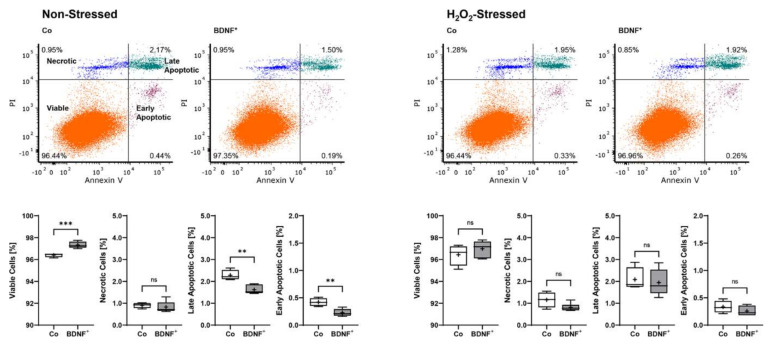
Apoptosis rate in BDNF-transfected ARPE-19 cells without or after H_2_O_2_-induced oxidative stress compared to non-transfected control cells. Non-transfected cells (Co, *n* = 8 cultures) and BDNF-transfected cells (BDNF^+^, transfected with 0.03 µg *SB100X* transposase and 0.47 µg *BDNF* transposon plasmid, *n* = 12 cultures) were seeded at a density of 1.2 × 10^5^ cells and cultivated for 96 h. Half of the cultures were treated with 150 µM H_2_O_2_ for an additional 24 h, then stained with annexin V and propidium iodide (PI) and analyzed by flow cytometry. Annexin V denotes early apoptotic cells, PI denotes necrotic cells, and double staining denotes late apoptotic cells. BDNF^+^ cultures had lower levels of apoptotic and necrotic cells and thus higher viability rates, although the differences were more pronounced in non-stressed ARPE-19 cell cultures (ns: not significant, ** *p* < 0.01, *** *p* = 0.0003, unpaired t test with Welch’s correction).

Non-stressed BDNF^+^ ARPE-19 cultures showed a lower apoptosis rate with a total of 2.67% non-viable cells divided into 0.23 ± 0.07% early apoptotic (EA), 1.62 ± 0.20% late apoptotic (LA), and 0.82 ± 0.26% necrotic (N) cells. This corresponded to a 25.8% lower value compared to non-stressed control cultures, which had a total of 3.60% non-viable cells divided into 0.42 ± 0.07% EA, 2.28 ± 0.23% LA, and 0.90 ± 0.12% N cells. BDNF^+^ cultures treated with 150 µM H_2_O_2_ for 24 h also exhibited a lower percentage of non-viable cells (3.02% total, divided into 0.26 ± 0.09% EA, 1.94 ± 0.60% LA, and 0.82 ± 0.18% N cells) than the non-transfected cultures treated with H_2_O_2_ (3.57% total, divided into 0.33 ± 0.11% EA, 2.08 ± 0.53% LA, and 1.16 ± 0.35% N cells), although less pronounced (Figure 4).

### 2.3. Neurite Outgrowth in SH-SY5Y Cells Co-Cultured with BDNF-Transfected ARPE-19 Cells

**Figure 5:**
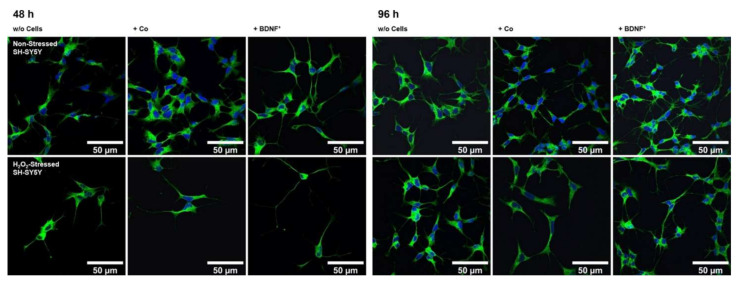
Neurite outgrowth in non-stressed and H_2_O_2_-stressed SH-SY5Y cells with or without co-cultivation of non-transfected and BDNF-transfected ARPE-19 cells. Representative confocal microscopy images of SH-SY5Y cells stained with neuron-specific beta-III tubulin antibodies (green) and DAPI (blue) are shown. Cells were cultivated for 48 or 96 h under the following conditions: non-stressed and H_2_O_2_-stressed SH-SY5Y alone (w/o Cells), non-stressed and H_2_O_2_-stressed SH-SY5Y with ARPE-19 cells (+ Co), non-stressed and H_2_O_2_-stressed SH-SY5Y with transfected ARPE-19 cells (+ BDNF^+^, transfected with 0.03 µg *SB100X* transposase and 0.47 µg *BDNF* transposon plasmid). Number and length of neurites were analyzed using the Simple Neurite Tracer plugin from Fiji-ImageJ.

Using an in vitro co-culture model, we demonstrated that BDNF^+^ ARPE-19 cells secrete sufficient amounts of recombinant BDNF to trigger neurite outgrowth and elongation in non-stressed and oxidatively stressed SH-SY5Y neuroblastoma cell cultures. Pretreatment of SH-SY5Y cells with 150 µM H_2_O_2_ for 24 h, to mimic an in vitro AMD disease model, induced oxidative stress through the generation of reactive oxygen species and resulted in reduced cell numbers compared to the non-stressed SH-SY5Y cell cultures (Figure 5). In co-cultures with BDNF^+^ ARPE-19 cells, BDNF was detected at concentrations of 927.1 ± 501.7 pg/mL and 2241 ± 1075 pg/mL after 48 h and 96 h, respectively. BDNF levels in co-cultures with non-transfected ARPE-19 cells were decreased 43- to 59-fold (21.50 ± 4.818 pg/mL at 48 h, 38.24 ± 9.664 pg/mL BDNF at 96 h). In SH-SY5Y cell cultures without co-cultivation, BDNF levels were 12.93 ± 0.973 pg/mL after 48 h and 13.55 ± 1.553 pg/mL after 96 h (data not shown).

**Figure 6:**
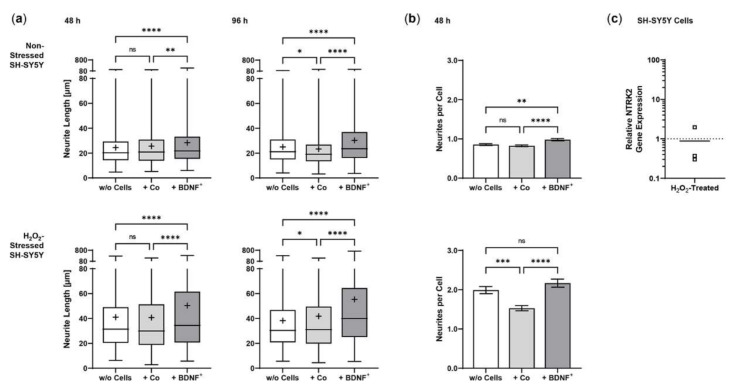
BDNF-mediated neurite outgrowth in non-stressed and H_2_O_2_-stressed SY-SY5Y cells after co-cultivation with ARPE-19 cells. Co-cultures were performed with different passages of BDNF-transfected cells (BDNF^+^, transfected 0.03 µg *SB100X* transposase and 0.47 µg *BDNF* transposon plasmid). In each experiment (*n* = 4), five images were analyzed for cultures with non-stressed SH-SY5Y cells and seven to ten images for cultures with H_2_O_2_-stressed SH-SY5Y cells. Neurite length was determined by converting pixels into µm (one pixel = 0.1 µm), excluding outgrowths of less than 2 µm. (**a**) Neurite lengths in the different cultures after 48 and 96 h. Non-stressed and H_2_O_2_-stressed SH-SY5Y cells co-cultivated with BDNF^+^ cells showed significantly increased neurite lengths than SH-SY5Y alone (w/o Cells) and SH-SY5Y co-cultivated with non-transfected cells (Co) (ns: not significant, ** *p* = 0.0041, **** *p* < 0.0001). Comparison of the two control cultures revealed significant differences only after 96 h (* *p* < 0.05, Brown-Forsythe and Welch ANOVA tests with Games-Howell’s multiple comparisons test). Number of values: 1158–1281 for non-stressed SH-SY5Y at 48 h, 1095–1369 for non-stressed SH-SY5Y at 96 h, 845–962 for H_2_O_2_-stressed SH-SY5Y at 48 h, 954–1550 for H_2_O_2_-stressed SH-SY5Y at 96 h. Data are presented as box and whisker plots (whiskers: minimum to maximum, mean values indicated by +). (**b**) Neurites per cell in the different cultures after 48 h. Significances were found for non-stressed SH-SY5Y cells co-cultivated with BDNF^+^ cells compared to both controls (ns: not significant, ** *p* = 0.0017, **** *p* < 0.0001). In H_2_O_2_-stressed SH-SY5Y cells, the differences were significant compared to the co-cultivation with Co cells (*** *p* = 0.0001, **** *p* < 0.0001, Brown-Forsythe and Welch ANOVA tests with Games-Howell’s multiple comparisons test). Number of values: 1181–1496 for non-stressed SH-SY5Y at 48 h, 444–553 for H_2_O_2_-stressed SH-SY5Y at 48 h. Data are presented as mean ± SEM. (**c**) *NTRK2* gene expression was analyzed by qPCR. Expression in SH-SY5Y cultures with H_2_O_2_ exposure (*n* = 3) was related to that in cultures without H_2_O_2_ exposure (*n* = 3), which was set to 1 (dashed line). The mean value of the H_2_O_2_-treated cultures was almost 1 (no significance, one sample t test).

Co-culturing SH-SY5Y cells with BDNF^+^ ARPE-19 cells had a positive effect on neurite outgrowth under both non-stress and oxidative stress conditions: after 48 h, non-stressed SH-SY5Y cells showed a mean neurite length of 28.37 ± 23.48 µm, whereas SH-SY5Y cells without ARPE-19 cells or co-cultured with non-transfected ARPE-19 cells showed a mean neurite length of 24.37 ± 15.37 µm and 25.58 ± 18.45 µm, respectively. After 96 h, the mean neurite length in SH-SY5Y cells co-cultured with BDNF^+^ cells further increased to 30.27 ± 21.54 µm, compared to 25.02 ± 14.62 µm (SH-SY5Y cells alone) and 23.35 ± 16.34 µm (SH-SY5Y plus non-transfected ARPE-19 cells, Figure 6a). Neurite outgrowth was more pronounced in the oxidatively stressed SH-SY5Y cell cultures and increased 1.5- to 1.8-fold compared to the non-stressed SH-SY5Y cultures. Again, co-cultivation with BDNF^+^ ARPE-19 cells showed the longest SH-SY5Y neurites (50.37 ± 49.08 µm after 48 h, 55.36 ± 54.92 µm after 96 h). The neurite lengths of SH-SY5Y cells without co-cultivation were 41.07 ± 35.07 µm and 38.23 ± 29.97 µm after 48 and 96 h, respectively. In SH-SY5Y cells co-cultured with non-transfected ARPE-19 cells, neurite lengths of 40.74 ± 33.20 µm (48 h) and 41.83 ± 37.18 µm (96 h) were measured (Figure 6a). Within the non-stressed SH-SY5Y cultures, the number of neurites per cell increased from 0.86 (without ARPE-19 cells) and 0.82 (with non-transfected ARPE-19 cells) to 0.98 when co-cultivated with BDNF^+^ ARPE-19 cells after a cultivation time of 48 h. For oxidatively stressed SH-SY5Y cell cultures, the corresponding values were 1.99 (without ARPE-19 cells), 1.53 (with non-transfected ARPE-19 cells), and 2.17 (with BDNF^+^ ARPE-19 cells, Figure 6b). However, after a cultivation time of 96 h, no increase in the number of neurites per cell was observed in either non-stressed or oxidatively stressed SH-SY5Y cells cultured with BDNF^+^ ARPE-19 cells (data not shown). In addition, SH-SY5Y cells showed no significant difference in the expression of the neurotrophic receptor tyrosine kinase 2 (*NTRK2*, encoding the TrkB receptor) gene when cultivated in the presence of H_2_O_2_ (Figure 6c).

### 2.4. Sleeping Beauty Transposon-Mediated Delivery of the Human BDNF Gene into Primary Human RPE Cells

**Figure 7:**
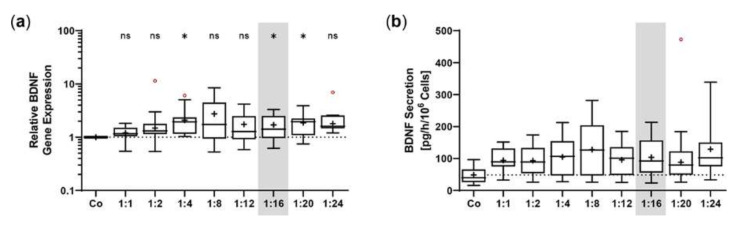
Transfection of primary hRPE cells using different ratios of *SB100X* transposase plasmid and *BDNF* transposon plasmid. In each experiment (*n* = 10), 1 × 10^5^ primary RPE cells from a total of eight donors (age: 71.9 ± 8.84 years, gender: five males and three females, time postmortem: 27.6 ± 16.3 h, cultivation time before transfection: 67.3 ± 24.0 days) were transfected with an increasing ratio (1:1–1:24) of *SB100X* transposase to *BDNF* transposon plasmid at a total concentration of 0.5 µg, plus one control without the addition of plasmid DNA (Co). Cultures were terminated 36.8 ± 10.5 days after transfection. Cells were used for isolation of total RNA. Medium used for ELISA was added to the cells in a defined volume 24 h before culture termination. (**a**) *BDNF* gene expression was analyzed by qPCR. Total *BDNF* expression in transfected cultures was related to the expression in Co cultures, which was set to 1 (dashed line). An increase in *BDNF* expression was observed in the transfected cultures (ns: not significant, * *p* < 0.05, one sample t test). (**b**) BDNF secretion was quantified by ELISA using precisely defined cell culture supernatants. Values of the transfected cultures were higher than those of the Co cultures, although not significantly (Brown-Forsythe and Welch ANOVA tests with Dunnett’s T3 multiple comparisons test). Data are presented as box and whisker plots (whiskers: minimum to maximum, mean values indicated by +). Outliers were evaluated using the ROUT method (Q = 1%, indicated by red open circles).

Transposition efficiency in primary hRPE cells was determined by *BDNF* expression and BDNF secretion for plasmid ratios of 1:1 to 1:24 at a constant total plasmid amount of 0.5 µg. *BDNF* expression increased with increasing plasmid ratio, although to a lesser extent than observed in ARPE-19 cells. The highest expression levels were determined at plasmid ratios of 1:2, 1:4, 1:8, and 1:24; however, the highest variation was also observed at these ratios. Plasmid ratios ranging from 1:12 to 1:20 showed expression values between 1.70 ± 0.95 (1:16) and 1.86 ± 0.93 (1:20, Figure 7a). The results of BDNF secretion were consistent with those of *BDNF* expression. ELISA-based quantification revealed a baseline secretion of 48.4 ± 27.7 pg BDNF/h/10^6^ cells in non-transfected hRPE cell cultures. In the transfected hRPE cell cultures, the mean BDNF secretion was approximately twice as high, with the lowest value of 93.37 ± 47.68 pg/h/10^6^ cells for the 1:2 ratio and the highest value of 128.9 ± 99.53 pg/h/10^6^ cells for the 1:24 ratio (Figure 7b). Subsequent experiments were performed with a plasmid ratio of 1:16 (0.03 µg *SB100X* transposase plasmid and 0.47 µg *BDNF* transposon plasmid), because the results for both *BDNF* expression and BDNF secretion varied more slightly.

**Figure 8:**
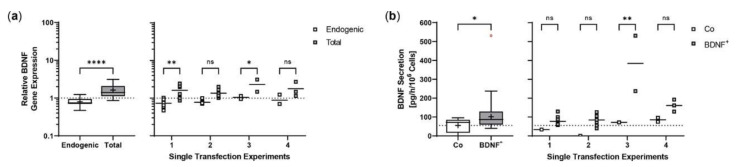
Transposition efficiency in primary hRPE cells transfected with a defined ratio of *SB100X* transposase to *BDNF* transposon plasmid. In each experiment (*n* = 4), two to eight transfections were performed using 5 × 10^4^ or 1 × 10^5^ cells from individual donors (donor age: 66.5 ± 9.95 years, gender: four males, time postmortem: 29.8 ± 28.0 h, cultivation time before transfection: 74.8 ± 51.1 days) and a mixture of 0.03 µg *SB100X* transposase and 0.47 µg *BDNF* transposon plasmid (BDNF^+^), plus one or two controls without the addition of plasmid DNA (Co). Cultures were terminated 22 days after transfection. Cells were used for isolation of total RNA. Medium used for ELISA was added to the cells in a defined volume 24 h before culture termination. (**a**) *BDNF* gene expression was analyzed by qPCR. Endogenous and total *BDNF* expression in BDNF^+^ cultures were related to the respective expression in Co cultures, which were set to 1 (dashed line). An increase in total *BDNF* expression was observed in the BDNF^+^ cultures (ns: not significant, summarized data: **** *p* < 0.0001, unpaired t test with Welch’s correction; single experiments: * *p* = 0.0418, ** *p* = 0.0023, 2way ANOVA with Šídák’s multiple comparisons test). (**b**) BDNF secretion was quantified by ELISA using precisely defined cell culture supernatants. Values of the BDNF^+^ cultures were higher than those of the Co cultures (ns: not significant, summarized data: * *p* = 0.0333, unpaired t test with Welch’s correction; single experiments: ** *p* = 0.0014, 2way ANOVA with Šídák’s multiple comparisons test). Data are presented as box and whisker plots (whiskers: minimum to maximum, mean values indicated by +) and as individual values per experiment (line at mean). Outliers were evaluated using the ROUT method (Q = 1%, as indicated by red open circles).

Repeated experiments with two to eight individual transfections and one or two non-transfected controls were performed with an initial number of 5 ×10^4^ or 1 × 10^5^ hRPE cells, depending on the number of cells available. Cultures were terminated 22 days after transfection to analyze *BDNF* gene expression and BDNF protein secretion in each approach. Transfected cells showed a 2-fold increase in total *BDNF* expression compared with endogenous *BDNF* expression, with no striking differences between the single experiments (Figure 8a). ELISA-based quantification of BDNF secretion almost matched the results observed for *BDNF* gene expression. Transfected cells secreted an average of 123.0 ± 108.5 pg BDNF/h/10^6^ cells into the medium, a 2.2-fold increase compared with 55.14 ± 38.07 pg/h/10^6^ cells for non-transfected cells. The highest increase was observed in experiment 3 with a mean BDNF secretion of 384.3 ± 207.9 pg/h/10^6^ cells for BDNF^+^ cells, which was 5.0-, 4.6-, and 2.4-fold higher than the mean BDNF secretion in experiments 1, 2, and 4, respectively (Figure 8b). After a cultivation period of seven months, long-term cultures of transfected primary hRPE cells showed a mean BDNF secretion of 140.5 pg/h/10^6^ cells, which was 1.8-fold higher than the secretion rate of the corresponding non-transfected cells (data not shown).

### 2.5. Neurite Outgrowth in SH-SY5Y Cells Co-Cultured with BDNF-Transfected Primary Human RPE Cells

**Figure 9:**
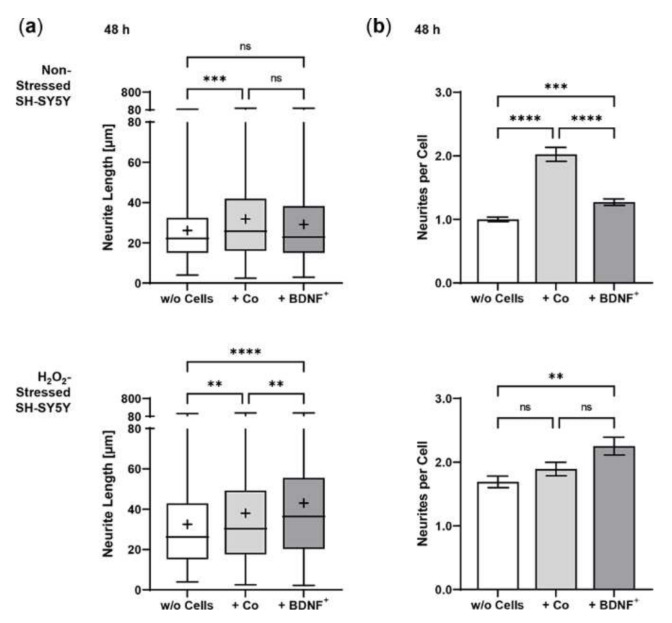
BDNF-mediated neurite outgrowth in non-stressed and H_2_O_2_-stressed SY-SY5Y cells after co-cultivation with primary hRPE cells. Co-cultures were performed with BDNF-transfected cells (BDNF^+^, transfected with 0.03 µg *SB100X* transposase and 0.47 µg *BDNF* transposon plasmid) from two donors (age: 65.5 ± 6.36 years, gender: one male and one female, time postmortem: 34.6 ± 18.2 h, cultivation time before transfection: 67.5 ± 0.71 days). Co-cultures were started 25 days after transfection. In each experiment (*n* = 2), five images were analyzed for cultures with non-stressed SH-SY5Y cells and seven to ten images for cultures with H_2_O_2_-stressed SH-SY5Y cells. Neurite length was determined after 48 h by converting pixels into µm (one pixel = 0.1 µm), excluding outgrowths of less than 2 µm. (**a**) Neurite lengths in the different cultures after 48 h. Non-stressed SH-SY5Y cells co-cultivated with non-transfected cells (Co) and BDNF^+^ cells showed increased neurite lengths compared to SH-SY5Y cells alone (w/o Cells) (*** *p* = 0.0001 for Co, not significant for BDNF^+^). Note that neurite lengths were more increased in Co co-cultures than in BDNF^+^ co-cultures, although not significantly. H_2_O_2_-stressed SH-SY5Y cells co-cultivated with Co and BDNF^+^ cells showed significantly increased neurite lengths compared to SH-SY5Y alone (** *p* = 0.0023, **** *p* < 0.0001). Furthermore, BDNF^+^ co-cultures showed significantly increased neurite lengths compared to Co co-cultures (** *p* = 0.0053, Kruskal-Wallis test with Dunn’s multiple comparisons test). Number of values: 690–815 for non-stressed SH-SY5Y, 580–613 for H_2_O_2_-stressed SH-SY5Y. Data are presented as box and whisker plots (whiskers: minimum to maximum, mean values indicated by +). (**b**) Neurites per cell in the different cultures. For non-stressed SH-SY5Y cells, significant differences were observed for all comparisons (*** *p* = 0.0005, **** *p* < 0.0001); however, the highest number of neurites per cell was observed for Co co-cultures. For H_2_O_2_-stressed SH-SY5Y cells, Co and BDNF^+^ co-cultures showed increased numbers of neurites per cell compared to SH-SY5Y alone (not significant for Co, ** *p* = 0.0013 for BDNF^+^, Ordinary one-way ANOVA with Tukey’s multiple comparisons test). Number of values: 341–781 for non-stressed SH-SY5Y, 259–343 for H_2_O_2_-stressed SH-SY5Y. Data are presented as mean ± SEM.

In co-cultures with BDNF^+^ hRPE cells, BDNF was detected at a concentration of 17.27 ± 1.984 pg/mL after 48 h, which was only slightly higher than in co-cultures with non-transfected hRPE cells (14.87 ± 1.662 pg/mL) and SH-SY5Y cells without co-culturing (12.56 ± 1.909 pg/mL, data not shown). Co-culturing with primary hRPE cells had a positive effect on neurite outgrowth of SH-SY5Y cells under both non-stress and oxidative stress conditions. Non-stressed SH-SY5Y cells without co-culturing showed a mean neurite length of 26.14 ± 15.97 µm compared to 31.81 ± 21.94 µm and 29.16 ± 20.00 µm for SH-SY5Y cells co-cultured with non-transfected and BDNF-transfected hRPE cells, respectively. However, neurite outgrowth was slightly more pronounced in the co-cultures with non-transfected cells than in the co-cultures with BDNF^+^ cells (Figure 9a). An overall 1.2- to 1.5-fold increase in neurite outgrowth was observed in the oxidatively stressed SH-SY5Y cultures. Here, co-culturing with BDNF^+^ hRPE cells showed the longest SH-SY5Y neurites (42.99 ± 31.98 µm), which were 32% and 13% longer than the neurites of SH-SY5Y cells without co-culturing (32.52 ± 24.49 µm) and co-cultured with non-transfected hRPE cells (38.01 ± 29.34 µm, Figure 9a), respectively. These results were also reflected in the number of neurites per cell. In oxidatively stressed SH-SY5Y cultures, an increase from 1.69 (without co-cultivation) and 1.89 (co-cultivated with non-transfected hRPE cells) to 2.25 (co-cultivated with BDNF^+^ hRPE cells) was observed. For non-stressed SH-SY5Y cultures, the corresponding values were 1.00 (without hRPE cells), 2.02 (with non-transfected hRPE cells), and 1.27 (with BDNF^+^ hRPE cells, Figure 9b).

## 3. Discussion

The non-viral *SB* transposon system enables efficient and sustained transgene transfer. It circumvents potential side effects associated with virus-based gene transfer, such as immunogenicity described for adenoviruses and the increased risk of insertional mutagenesis characterizing retroviruses and lentiviruses. The hyperactive *SB100X* transposase ensures efficient and stable integration of the *BDNF* transgene-encoding transposon into the genome of RPE cells. However, excessive amount of *SB100X* transposase decreases transposition efficiency [49]. For this reason, we needed to optimize the transposase-to-transposon ratio. In ARPE-19 cells, *BDNF* gene expression and BDNF protein secretion continuously increased. This increase reached a steady state at a ratio of 1:16, corresponding to concentrations of 0.03 µg *SB100X* transposase plasmid and 0.47 µg *BDNF* transposon plasmid. These results were comparable to those for the *PEDF* transposon plasmid from our previous studies, in which PEDF secretion was approximately constant over a range of 1:16 to 1:36 [24]. However, in primary hRPE cells, the increase in the ratio of *SB100X* transposase to *BDNF* transposon was not as obvious. Here, the steady state was already reached at a ratio of 1:8, and the decision to use the ratio of 1:16 for subsequent experiments was based on the results with the lowest standard deviation. In another study, a cytotoxic effect was demonstrated with high and long-lasting expression of the *SB* transposase. This effect, however, could be avoided by only short-term and dose-controlled transient expression of the transposase [50]. Our approach also involves dose-regulated and transient expression of the *SB100X* transposase, which we have already shown in previous studies to be undetectable at the latest 28 days after transfection [24].

In contrast to retroviruses and lentiviruses or *piggyBac* transposon-based genomic integration [51], *SB100X*-mediated integration is nearly random and shows no increased tendency to integrate into coding genomic segments. We previously reported a similar result for the integration profile of pigment epithelial cells [22,25], suggesting that the close-to-random integration is reproducible. The average transposon copy numbers of the BDNF-transfected ARPE-19 cell cultures were lower than those described for *SB*-mediated transfection of CAR T cells [52]. The values of the individual transfected ARPE-19 cultures were different (minimum: 0.65; maximum: 1.76), which could be due to the number of genomically integrated *BDNF* gene copies or the percentage of cells initially transfected. We previously showed that *SB100X*-based transfer of the *PEDF* transgene into ARPE-19 cells resulted in an initial transfection efficiency of 24% to 32% [24].

The *SB* transposon system was introduced into the RPE cells by electroporation. The outcome of electroporation, i.e., cell viability and transfection efficiency, depends on several parameters, such as the strength of the electric field and pulse duration as well as the composition and conductivity of the electroporation buffer [53,54]. Sherba and colleagues showed that Mg^2+^-based buffers increased cell viability. They hypothesized that magnesium has an effect on viability after electroporation due to its role in activating ATPase membrane ion channels [55]. In our study, the components of buffer R used for the Neon Transfection System are not known. However, in terms of clinical application, an electroporation buffer with a known composition is mandatory. Higher transfection efficiency can be achieved by increasing electroporation parameters, but this is associated with cell loss, i.e., lower cell survival after transfection [56,57,58]. Repeated electroporation could also lead to higher transgene expression. In addition to the stress caused by a second electroporation, it may also require additional passaging of the cells. Repeated passages are not beneficial for the uniform morphology of primary RPE cells, as they lead to pigment loss, a less pronounced hexagonal shape, multi-layering, and variations in cell size [59]. Another option is to use more plasmid DNA, but this is also critical, both in terms of cell viability [56,60] and clinical application. Excess plasmid DNA that is not integrated must be removed from the genetically modified cells prior to their use in patients. A fourth option is to use smaller plasmids, as transfection efficiency is inversely proportional to plasmid size [61,62]. One example is the plasmid free of antibiotic resistance markers (pFAR), which was shown to double the number of transfected cells and more than double the amount of secreted PEDF when combined with *SB100X* transposase [26]. In addition to higher efficiency, the pFAR miniplasmid system offers an improved safety profile; by eliminating the antibiotic resistance gene normally required for efficient plasmid production, some safety risks in clinical use are avoided.

BDNF used in this study supports neuronal cell differentiation and maturation and promotes neuronal survival and growth [63,64], possibly through interaction with the Wnt/β-catenin signaling pathway mediated by glycogen synthase kinase-3 beta (GSK-3β) [65]. Furthermore, the neuroprotective effect of BDNF was also demonstrated in vivo in hypoxic ischemic brain injury [66,67]. In addition to its effects on neuronal cells, BDNF increased proliferation and expression of cell cycle regulatory proteins in porcine uterine luminal epithelial cells [68], promoted proliferation and expression of proliferation-related genes in bovine granulosa cells [69], and supported regeneration of alveolar epithelium [70]. We observed that BDNF^+^ ARPE-19 cultures had higher proliferation and cell viability rates than non-transfected cultures, whereas treatment with H_2_O_2_ resulted in a lower proliferation rate. A possible explanation for this could be the boosted metabolism of BDNF-transfected cells in the context of increased expression and secretion of BDNF (see Figure 3d). Ogura and colleagues showed that subtoxic levels of H_2_O_2_ (treatment with 1-1000 µM for 30 min) induced *BDNF* expression in rat pheochromocytoma (PC12) cells [71]. With regard to transplantation of the transfected cells into the eye as a continuous BDNF delivery system, increased proliferation is undesirable and would raise biosafety concerns. It is important to note that this increased proliferation was only observed in the metabolically active ARPE-19 cells, but not in the actual cells to be transplanted, namely the less metabolically active primary hRPE cells.

The overall goal of our gene therapy approach is to protect neuronal/sensory cells in patients suffering from degenerative retinal diseases such as dry AMD. The characteristics of dry AMD range from lipofuscin deposition and drusen formation to degeneration of photoreceptors and the RPE triggered by immune activation, inflammation, and oxidative stress. These mechanisms are all linked to the RPE. Many different therapeutic approaches are currently being pursued, including antioxidants, anti-inflammatory agents, complement inhibitors, retinal implants, and gene and cell-based therapies [72]. The latter aim not only to replace the degenerated RPE but also to support the surrounding cells and tissues. Nowadays, these therapies mainly focus on the transplantation of stem cell-derived RPE cells, which are used in early stages of clinical trials. Their use can overcome earlier challenges, such as providing sufficient quantities of RPE cells (reviewed in [73]). Additional expression of BDNF in the eye is expected to exert protective effects. In several in vivo models, BDNF was already shown to protect RGCs and reduce vision loss [74,75]. BDNF and its receptor TrkB are naturally expressed in RPE cells [76] and play a critical role in RPE differentiation and survival [77]. Inana and colleagues recently demonstrated that phagocytic function was significantly reduced in primary RPE cultures from AMD donor eyes and that BDNF could restore this dysfunction [78]. Patients with dry AMD had lower aqueous humor BNDF concentrations, which correlated with a decrease in outer retinal thickness [43]. In another study of patients with dry AMD, thinning of the RGC layer was observed [79]. However, neurons in human retinas with dry AMD are able to remodel and form new synaptic complexes by sprouting processes [80].

Neuroprotection aims to reduce apoptosis in order to halt the progression of GA. In our approach, this is achieved by transplanting BDNF-transfected cells into the eye, more specifically into the subretinal space. The fact that the highest number of SH-SY5Y cells was found in the BDNF^+^ approaches at the end of co-cultivation with H_2_O_2_ exposure may indicate that the secreted BDNF protects the SH-SY5Y cells from apoptosis. However, this effect was not observed after a cultivation time of 48 h or in the approaches without H_2_O_2_ treatment (data not shown). To analyze the neuroprotective function of BDNF-transfected cells, we used an adapted version of the in vitro bioassay model described by Chen and Foldvari [81]. Transfected RPE cells were co-cultured with SH-SY5Y cells, a human neuroblastoma cell line that was used to study neurodegenerative diseases such as PD and AD [82,83]. Since oxidative stress contributes to the pathological processes of AMD, SH-SY5Y cells were pre-incubated with culture medium containing H_2_O_2_ to mimic oxidatively damaged neuronal cells. Initial experiments showed that approximately 50% of cultured SH-SY5Y cells survived at a concentration of 150 µM H_2_O_2_ (LC50). This reduced cell number resulted in increased SH-SY5Y neurite outgrowth in the co-culture experiments with H_2_O_2_ than in the co-culture experiments without oxidative stress. Expression of the receptor TrkB, which is required for BDNF action, was not affected by this H_2_O_2_ treatment (see Figure 6c).

Regarding apoptosis, a higher number of annexin V/propidium iodide (PI)-stained cells, and an increased BAX/BCL2 ratio are known biochemical indicators. H_2_O_2_ is a commonly used agent to induce these processes [84,85], which in turn can be counteracted by neuroprotective factors. In the absence of H_2_O_2_, BDNF^+^ ARPE-19 cell cultures already showed significantly reduced numbers of early and late apoptotic cells, with a discrepancy to the values of non-transfected control cultures in the range of 1%. The protective effect of BDNF was also observed after treatment with 150 µM H_2_O_2_ but was not as pronounced. This could be due to the robustness of the ARPE-19 cell line and the relatively low H_2_O_2_ concentration, which was, however, chosen analogously to the other experiments. In addition, H_2_O_2_-treated BDNF^+^ ARPE-19 cultures showed an increased *BAX*/*BCL2* gene expression ratio immediately after H_2_O_2_ exposure, which decreased with increasing cultivation time, whereas in non-treated BDNF^+^ cultures the *BAX*/*BCL2* gene expression ratio slightly increased. This opposite effect could be explained by the continuously increased *BDNF* expression detected in BDNF^+^ cultures after H_2_O_2_ exposure.

In both non-stressed and H_2_O_2_-stressed SH-SY5Y cells, significant prolongation of neurites occurred after 48 and 96 h of co-cultivation with BDNF^+^ ARPE-19 cells. Here, we demonstrated that BDNF exhibits neuroprotective effects at concentrations as low as approximately 1 ng/mL. Other studies described effects on neurite outgrowth at BDNF concentrations ranging from 3.75 to 10.4 ng/mL [81] as well as 50 ng/mL [86], although in the latter study the experiments were performed in serum-free medium. We also determined the number of neurites and related it to the number of SH-SY5Y cells. We found that after 48 h of cultivation, the number of neurites per cell was significantly increased in co-cultures with BDNF^+^ ARPE-19 cells. However, this effect decreased and was no longer observed after 96 h of co-culture (data not shown). A possible explanation could be that BDNF first promotes the formation of neurites and then their length growth. The culture medium was not changed during co-cultivation, so the amount of available nutrients decreased while metabolites accumulated. This may also have resulted in the promotion of length growth of existing neurites rather than the formation of new neurites. In primary hRPE cells, significantly increased neurite outgrowth was observed only in the co-cultures of BDNF^+^ cells with H_2_O_2_-stressed SH-SY5Y cells. This is apparently due to the low concentration of secreted BDNF, which was only slightly higher than in the corresponding control cultures. Based on the results of BDNF^+^ ARPE-19 cells, a more pronounced neuroprotective effect can be assumed for primary hRPE cells when transfection efficiency is increased, because transfection efficiency correlates with the amount of BDNF secreted. Despite all this, careful determination of the appropriate protein concentration is essential to ensure an effective and long-term effect of *BDNF* gene therapy. In addition to mature BDNF, pro-BDNF was also detected in the culture supernatants. Pro-BDNF has the opposite effect to mature BDNF: it induces apoptosis and inhibits proliferation [87,88,89]. Moreover, several studies have shown that excessive BDNF concentration led to downregulation of the high-affinity receptor TrkB [90,91,92]. However, another study in a rat glaucoma model showed that moderate overexpression of BDNF restored normal TrkB levels and did not cause downregulation [48].

The results show that *SB100X*-transfected RPE cells secrete increased amounts of BDNF. The BDNF-transfected cells are functional: their protective effect was demonstrated by increasing the BAX/BCL2 ratio, decreasing the apoptosis rate, and inducing neuronal outgrowth. This study is an important first step toward the development of a pigment epithelial cell-based *BDNF* gene therapy that could be used as an advanced therapy medicinal product (ATMP) for the treatment of neurodegenerative eye diseases. ATMPs are a novel class of innovative and complex biological products. Positive non-clinical results are an important prerequisite for their further development towards a clinically applicable and compliant product. Our next steps are to transfer the functional assays to a retina-related experimental setup, such as an ex vivo retinal organ co-culture model, and to characterize the transfected primary human RPE cells immunohistochemically before using them in appropriate transplantation experiments, e.g., to investigate the molecular mechanism of the effects of BDNF in vivo.

## 4. Materials and Methods

### 4.1. Plasmid Constructs

**Figure 10:**
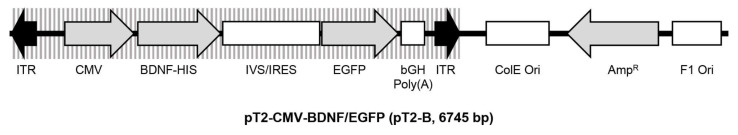
Schematic drawing of the transposon plasmid pT2-CMV-BDNF/EGFP. The plasmid is based on pT2/HB and has a size of 6745 base pairs. The expression cassette is flanked by inverted terminal repeats (ITRs) and contains a human *BDNF* cDNA expressed under control of the CMV promoter and fused to a 6x histidine-tag (HIS), followed by a pIRES-EGFP-vector-derived synthetic intron (IVS) and an internal ribosomal entry site (IRES) which enable co-translation of BDNF and the enhanced green fluorescence protein (EGFP).

The pT2-CMV-BDNF/EGFP transposon plasmid (pT2-B, Figure 10) is based on pT2/HB (Addgene plasmid #26556). The eukaryotic expression cassette is flanked by inverted terminal repeats (ITRs) and contains the cDNA of the human *BDNF* gene (Ensembl Gene ID: ENSG00000176697), which is under control of the cytomegalovirus (CMV) promoter and fused to a C-terminal 6× histidine-tag. Co-translation of the enhanced green fluorescent protein (EGFP) is enabled by the pIRES-EGFP-vector-derived synthetic intron (IVS) and an internal ribosomal entry site (IRES) [93]. First, a BDNF-including sequence was amplified from the cDNA of primary hRPE cells (bdnf-F: 5′-CAT TGA GCT CGC TGA AGT TGG CTT CCT AGC-3′ and bdnf-R: 5′-ACT GTT TCC CTT CTG GTC ATG GAC ATG TCC-3′). The PCR fragment was purified using the QIAquick PCR Purification Kit (Qiagen, Hilden, Germany) according to the manufacturer’s protocol and applied in a second PCR for amplification of the BDNF cDNA extended by the sequences for the restriction enzymes *Nhe*I (n-nhe-bdnf: 5′-caa gct ggc tag cca ccA TGA CCA TCC TTT TCC TTA CTA TGG-3′) and *Not*I (c-not-bdnf: 5′-atg ggg ccc tgc ggc cgc TCT TCC CCT TTT AAT GGT CAA TGT AC-3′). The purified PCR fragment was introduced into the pT2-CMV-PEDF/EGFP transposon plasmid [24] via *Nhe*I/*Not*I-mediated cloning. The resulting pT2-B transposon plasmid was propagated in an *E. coli* K-12 strain, purified using appropriate plasmid purification kits (Qiagen, Hilden, Germany), and characterized by sequencing (Eurofins Genomics, Ebersberg, Germany).

### 4.2. Cell Line Cultivation

ARPE-19 cells (ATCC CRL-2302) were maintained in Dulbecco’s Modified Eagle’s Medium (DMEM)/F12 (PAN-Biotech, Aidenbach, Germany) supplemented with 10% fetal bovine serum (FBS; PAN-Biotech, Aidenbach, Germany), 80 U/mL penicillin and 80 µg/mL streptomycin (Pen/Strep; Merck, Darmstadt, Germany), and 2.5 µg/mL amphotericin B (AmphoB; Merck, Darmstadt, Germany) at 37 °C in a humidified atmosphere of 95% air and 5% CO_2_. Medium was changed twice a week. Cells were passaged weekly using 0.05% trypsin-EDTA (Thermo Fisher Scientific, Waltham, MA, USA) at a ratio of 1:10.

SH-SY5Y cells (ATCC CRL-2266) were maintained in a 1:1 ratio of Eagle’s Minimum Essential Medium (EMEM; LGC Standards, Wesel, Germany) and Ham’s F12 (PAN-Biotech, Aidenbach, Germany) supplemented with 10% FBS and Pen/Strep at 37 °C in a humidified atmosphere of 95% air and 5% CO_2_. Medium was changed twice a week. Cells were passaged biweekly using 0.05% trypsin-EDTA and seeded at a density of 12,500 cells/cm^2^.

### 4.3. Isolation and Cultivation of Primary Human RPE Cells

Human eyes from 15 donors (67.4 ± 10.6 years of age, nine males and six females) were obtained from the Aachen Cornea Bank (Department of Ophthalmology, University Hospital RWTH Aachen). Eyes were removed 29.9 ± 17.3 h postmortem, after informed consent was obtained in accord with the Declaration of Helsinki protocols. The procedures for the collection and the use of human samples were approved by the Ethics Committee at the RWTH Aachen Faculty of Medicine (reference number: EK 290/18). Isolation of primary hRPE cells was performed as previously described [94]. Briefly, after separation of the anterior segment, vitreous and retina were removed. The posterior eyecup was filled twice with 1 ml DMEM/F12 supplemented with 10% FBS, Pen/Strep, and AmphoB. Cells were harvested twice by gently brushing the RPE with a fire-polished curved glass Pasteur pipette and transferred into a Petri dish. The cell suspension of each eye globe was carefully resuspended and seeded into three wells of a 24-well cell culture plate, each filled up to 1 ml with DMEM/F12 supplemented with 10% FBS, Pen/Strep, and AmphoB. Primary hRPE cell cultures were maintained at 37 °C in a humidified atmosphere of 95% air and 5% CO_2_ until confluence was reached. Medium was changed twice a week.

### 4.4. Electroporation of ARPE-19 Cells and Primary Human RPE Cells

Electroporations were performed with the Neon Transfection System using the 10 µl Kit (Thermo Fisher Scientific, Waltham, MA, USA) as already described [24,25]. For ARPE-19 cells, two pulses with a voltage of 1,350 V and a width of 20 ms were applied. For primary hRPE cells, two pulses of 1,100 V and 20 ms were used. 1 × 10^5^ ARPE-19 cells or 5 × 10^4^ primary hRPE cells were resuspended in 11 µl buffer R and combined with 2 µl of purified plasmid mixture containing the *SB100X* transposase plasmid and the *BDNF* transposon plasmid at a total concentration of 0.5 µg. Each experiment also included controls with electric field application but without plasmid DNA. Transfected ARPE-19 cells were released into 6-well cell culture plates containing 3 ml of DMEM/F12 supplemented with 10% FBS, but without antibiotics and antimycotics. Transfected primary hRPE cells were transferred into 48-well cell cultures plates containing 0.5 ml of DMEM/F12 supplemented with 10% FBS. Pen/Strep and AmphoB were added with the first medium exchange three days after electroporation.

### 4.5. SDS-PAGE and Western Blot Analysis

BDNF secretion was assayed in control and BDNF-transfected cell cultures. For ARPE-19 cells, 10 µl of supernatant was directly analyzed. For primary hRPE cells, His-tagged BDNF was purified by Ni-NTA metal-affinity chromatography (Qiagen, Hilden, Germany) as already described [24]: 900 µl of supernatant was mixed with 30 µl of 50% Ni-NTA slurry and 300 µl of 4x incubation buffer. After agitation for 1 h at room temperature (RT), Ni-NTA resin was pelleted by centrifugation, washed twice with 1x incubation buffer, incubated with 30 µl of elution buffer for 20 min at RT, and again pelleted by centrifugation. Supernatants were mixed with an equal volume of 2x SDS sample buffer [95], heated for 5 min at 95 °C, and separated on a 15% SDS-polyacrylamide gel or a Mini-PROTEAN TGX Stain-Free Protein Gel (Any kD; Bio-Rad Laboratories, Hercules, CA). Stain-Free imaging enabled detection of the total protein content after electrophoresis using the ChemiDoc Imaging System together with the Image Lab software 6.1.0 (Bio-Rad Laboratories, Hercules, CA, USA). After transfer onto a 0.45 µm pore size nitrocellulose membrane (Merck) using the semi-dry transfer system (Bio-Rad Laboratories, Hercules, CA), blots were blocked with 3% BSA/Tris-buffered saline (TBS) for 1 h at RT, incubated overnight at 4 °C with anti-BDNF antibodies (rabbit polyclonal, 1:500 diluted in 3% BSA/TBS; Abcam, Cambridge, UK), followed by 1 h at RT with horseradish peroxidase-conjugated anti-rabbit antibodies (goat polyclonal, 1:2000 diluted in 10% milk powder/TBS; Abcam, Cambridge, UK). BDNF bands were visualized by chemiluminescence and evaluated using the ChemiDoc Imaging System or the LAS-3000 Imaging System (FujiFilm, Tokyo, Japan) together with the open-source image processing program ImageJ (Rasband, W.S., ImageJ, U.S. NIH, https://imagej.nih.gov/ij/index.html (accessed on 26 August 2022)).

### 4.6. BDNF ELISA

BDNF secretion was quantified in cultures of control and BDNF-transfected cells after incubation for a defined time in a defined volume of medium. Supernatants were analyzed using the Human Free BDNF Quantikine ELISA Kit (R&D Systems, Minneapolis, MN) according to the manufacturer’s protocol. For quantification as a function of the cell number, cells were trypsinized with 0.05% trypsin-EDTA and counted. Counting of ARPE-19 cells was performed using the CASY Cell Counter TT (Roche, Basel, Switzerland). Primary hRPE cells were mixed with trypan blue solution and counted using a hemocytometer.

### 4.7. Real-Time Quantitative PCR

Total RNA was isolated using the RNeasy Mini Kit together with the RNase-free DNase Set (Qiagen, Hilden, Germany). Reverse transcription was performed using the Reverse Transcription System (Promega, Madison, WI, USA) according to the manufacturer’s protocol. Real-time quantitative PCR (qPCR) was performed on a LightCycler 1.2 Instrument using the LightCycler FastStart DNA Master SYBR Green I kit (Roche, Basel, Switzerland) according to the manufacturer’s recommendations. For RPE cells, cDNA samples were run in duplicate using the primers endBDNF (F: 5′-CTT CTT GTG TAT GTA CAT TGA CC-3′, R: 5′-CTT AAC TGA ATA ATT TAC CCT GTT ATG-3′; RefSeq: NM_170735.6), totalBDNF (F: 5′-CGT GTG TGA CAG TAT TAG TGA GTG G-3′, R: 5′-GAC CTT TTC AAG GAC TGT GAC C-3′), BAX (F: 5′-GGG GAG CAG CCC AGA GG-3′, R: 5′-CGA TCC TGG ATG AAA CCC TGA-3′; RefSeq: NM_138761.4), and BCL2 (F: 5′-GTG TGT GGA GAG CGT CAA CC-3′, R: 5′-TAC AGT TCC ACA AAG GCA TCC CAG-3′; RefSeq: NM_000633.3). For SH-SY5Y cells, cDNA samples were run in triplicate using the primers NTRK2 (F: 5′-TGG ATG CAT ATC GTG CTC CG-3′, R: 5′-GTG CTT GGT TCA GCT CTT GC-3′ [96]; RefSeq: NM_001291937.2). Glyceraldehyde-3-phosphate dehydrogenase (GAPDH; F: 5′-ATC CCA TCA CCA TCT TCC AG-3′, R: 5′-ATG AGT CCT TCC ACG ATA CC-3′; RefSeq: NM_002046.3) was used as internal control gene. Reactions were performed using diluted cDNA and a primer concentration of 0.25 µM (0.50 µM for NTRK2). Thermal cycler conditions were as follows: initial denaturation at 95 °C for 10 min, followed by 50 cycles of denaturation at 95 °C for 10 s, annealing at 62 °C for 7 s (60 °C/8 s for NTRK2), and elongation at 72 °C for 15 s. Melting curve analysis confirmed the amplification specificity of each primer pair. Data were processed with LightCycler software 5.0 (Roche, Basel, Switzerland) and analyzed using the comparative CT (2^−ΔΔCT^) method, which describes relative gene expression [97].

### 4.8. Droplet Digital PCR

Genomic DNA (gDNA) was isolated using the QIAamp DNA Mini Kit (Qiagen, Hilden, Germany) according to the manufacturer’s protocol for cultured cells. To get rid of the transposon donor plasmids that did not serve as substrates for the transposition reactions and were therefore present as plasmid DNA in the gDNA samples, we digested 0.15 µg of gDNA with 20 units of *Dpn*I (New England Biolabs, Ipswich, MA, USA)—specific for *Dam* methylated DNA—overnight. To ensure uniform distribution of DNA molecules in the droplets, the magnetic bead-purified DNA was fragmented with *Cvi*QI (New England Biolabs, Ipswich, MA, USA) at RT for 2 h. 2 µl of the 30 µl digest was used for ddPCR with ddPCR Supermix for Probes (No dUTP) (Bio-Rad Laboratories, Hercules, CA, USA) according to the manufacturer’s recommendations. Primers and probes specific for the right inverted repeat of the SB transposon were: 5′-GAA TGT GAT GAA AGA AAT AAA-3′, 5-AGT TTA CAT ACA CCT TAG CC-3′, and RIR_FAM_BH1 5′FAM-TGG TGA TCC TAA CTG ACC TAA GAC AGG-BHQ1-3′. The human genome was quantified by a single copy gene (*RPP30*)-specific amplicon using the following primers: 5′-CTG TCT CCA CAA GTC CGC-3′, 5′-GGT TAA CTA CAG CTC CCA GC-3′, and the probe 5′-HEX-TGG ACC TGC GAG CGG GTT CTG ACC-BHQ1-3′. The cycling conditions for multiplex PCR were as follows: 95 °C for 10 min, 40 cycles of 94 °C for 30 s, 53 °C for 30 s, and 60 °C for 40 s, and 88 °C for 10 min. Droplet generation and counting were performed using the QX200 Droplet Digital PCR System (Bio-Rad Laboratories, Hercules, CA, USA). Copy number analysis was performed using QuantaSoft Analysis Pro software (Bio-Rad Laboratories, Hercules, CA, USA).

### 4.9. Cell Proliferation Assay

Non-transfected ARPE-19 cells, ARPE-19 cells exposed to the electric field without plasmid DNA, and BDNF-transfected ARPE-19 cells were seeded into 6-well cell culture plates at an initial density of 2 × 10^4^ cells. Medium was changed daily, and cell proliferation was analyzed at the indicated time points. To analyze cell proliferation under oxidative stress conditions, ARPE-19 cells were exposed to 150 µM H_2_O_2_ for 24 h one day after starting the cultures. Thereafter, the medium containing H_2_O_2_ was replaced with normal medium, and cell proliferation was analyzed at the indicated days. Cells were trypsinized with 0.05% trypsin-EDTA and counted using the CASY Cell Counter TT. Cell viability was determined by the CellTiter-Glo Luminescent Cell Viability Assay (Promega, Madison, WI, USA) according to the manufacturer’s protocol.

### 4.10. Annexin V/Propidium Iodide Assay

The rate of apoptosis was determined in ARPE-19 cells using the APC Annexin V Apoptosis Detection Kit with PI (BioLegend, San Diego, CA, USA). The assay is based on fluorochrome-labeled annexin V and propidium iodide (PI) to distinguish between early apoptotic, late apoptotic, and necrotic cells. Non-transfected and BDNF-transfected ARPE-19 cells were seeded into 24-well cell culture plates at a density of 1.2 × 10^5^ cells. After 96 h, cells were either treated with 150 µM H_2_O_2_ for 24 h or left untreated. Cells were stained as described in the manufacturer’s protocol and analyzed using the BD FACSCanto II flow cytometer along with BD FACSDiva software v9.0 (BD, Franklin Lakes, NJ, USA).

### 4.11. Co-Culture Model and Neurite Outgrowth Quantitation

Co-cultivation of transfected ARPE-19 cells with SH-SY5Y cells was adapted based on the two-layer, contact-independent bioassay model described by Chen and Foldvari [81]. ARPE-19 cells at 15,000/insert were seeded into 1.0-µm polyethylene terephthalate 24-well hanging cell culture inserts (Merck, Darmstadt, Germany) and cultivated in appropriate cell culture plates for 48 h. At the same time, SH-SY5Y cells at 80,000/well were cultivated in separate 24-well cell culture plates on 12 mm glass coverslips pre-coated with human recombinant biolaminin (BioLamina, Sundbyberg, Sweden) at 0.55 μg/cm^2^ for 48 h (non-stressed cells) or for 24 h followed by incubation with medium containing 150 µM H_2_O_2_ for another 24 h (oxidatively stressed SH-SY5Y cells). Thereafter, cell culture media were removed and the ARPE-19 containing inserts were transferred to the wells containing the SH-SY5Y cells. Co-cultures were initiated using defined volumes (inserts: 0.5 mL; wells: 1.0 mL) of a 1:1 ratio of EMEM and Ham’s F12 supplemented with 10% FBS and Pen/Strep and maintained for 48 h or 96 h. SH-SY5Y cells alone and co-cultures containing non-transfected ARPE-19 cells were used as controls.

**Figure 11:**
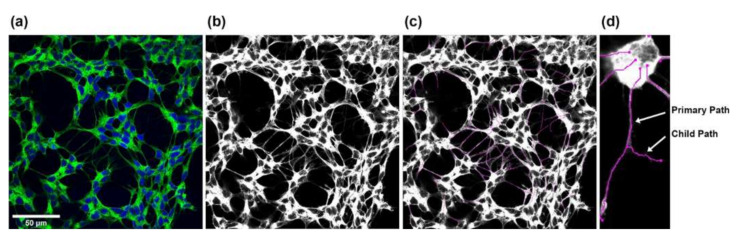
Steps for measuring SH-SY5Y neurite outgrowth. (**a**) RGB overlay images were merged from the individual monochrome images. (**b**,**c**) Starting from the corresponding grayscale images, all neurites in an image were measured using the Simple Neurite Tracer plugin from Fiji-ImageJ. (**d**) The total length of the neurites was determined by adding the main trunk (primary path) and the outgoing branches (child paths).

SH-SY5Y neurite outgrowth was evaluated by fixing the cells with 4% paraformaldehyde in phosphate buffered saline (PBS; 137 mM NaCl, 1.5 mM KH_2_PO_4_, 8.3 mM Na_2_HPO_4_, 2.7 mM KCl, pH 6.8) for 20 min at RT. After blocking with 10% FBS, 1% bovine serum albumin, and 0.1% Triton X-100 in PBS (pH 7.1) for 40 min at RT, 1x NorthernLights 493-conjugated mouse monoclonal anti-neuron-specific beta-III tubulin antibodies (R&D Systems, Minneapolis, MN, USA) in PBS were added for 3 h at RT in the dark. Cells were counterstained with 1 µg/mL 4′,6-diamidino-2-phenylindole (DAPI; Merck, Darmstadt, Germany) in PBS for 15 min at RT in the dark. Imaging was performed using a Nikon A1-Ti2-N-STORM confocal laser scanning microscope (Nikon Europe, Amsterdam, Netherlands) with ×63 oil immersion objective. Five pictures (cultures with non-stressed SH-SY5Y cells) and seven to ten pictures (cultures with oxidatively stressed SH-SY5Y cells) of 2048 × 2048 pixels up to 5530 × 5530 pixels for large neurons were captured from the four replicates of each culture condition after 48 h and 96 h. Images were converted to TIF format using NIS-Elements Viewer (Nikon Europe, Amsterdam, The Netherlands). Monochrome images were created for each channel and merged into RGB overlay images scaled to 8 bits (Figure 11a). To measure neurites, 8-bit grayscale images (Figure 11b) were analyzed using Simple Neurite Tracer [98], a free software plugin distributed by Fiji-ImageJ (https://imagej.net/software/fiji/ (accessed on 26/8/2022)), by defining the beginning–starting from the outer line of the nucleus–and the end of each neurite. The software calculated the length of the neurite by tracing a path between the two points (Figure 11c). Neurite length was displayed in pixels and converted to µm using a ratio of 0.1 µm per pixel, with the main trunk and outgoing branches for each neurite added up to the total length of the neurite (Figure 11d). This procedure was performed for all neurites in an image. Note that neurite outgrowths of less than 2 µm were not considered. The Cell Counter plugin (Fiji-ImageJ, https://imagej.net/software/fiji/ (accessed on 26 August 2022)) was used to determine the number of cells per image. All images were processed using the same settings.

### 4.12. Statistical Analysis

Statistical analyses were performed according to the tests indicated within the figure legends using GraphPad Prism software 9.1.1 (GraphPad, La Jolla, CA, USA) or IBM SPSS Statistics software 27.0 (IBM, Armonk, NY, USA).

## Data Availability

The raw data supporting the conclusions of this article will be made available by the authors upon request.
